# Inflation bald als Folgeeffekt der COVID-19-Pandemie?

**DOI:** 10.1007/s41025-021-00213-8

**Published:** 2021-03-02

**Authors:** Edoardo Beretta

**Affiliations:** Facoltà di Scienze economiche, Istituto di Economia Politica, Università della Svizzera italiana, Lugan, Schweiz

**Keywords:** COVID-19-Pandemie, Inflation, Lebenshaltungskosten, Liquidität, Wirtschaftspolitik, Costs of living, COVID-19 pandemic, Inflation, Liquidity, Economic policy

## Abstract

Obwohl man im Zuge der COVID-19-Pandemie aufgrund des dramatischen Einbruchs aller Wirtschaftsprognosen weltweit bislang von rezessiv-deflationären Auswirkungen ausgegangen ist, lassen sich Inflationsrisiken keineswegs ausschließen. Welche Folgeeffekte kann die Kombination aus milliardenhohen Liquiditätsspritzen bei starkem Produktionsrückgang sowie streng einzuhaltenden (und ebenso kostenträchtigen) Sicherheits- und Schutzauflagen entfalten? Und wieso sollte aus makroökonomischer Sicht zwischen „Inflation“ und „steigenden Lebenshaltungskosten“ unterschieden werden? Im vorliegenden Beitrag wird mithilfe eines logisch-analytischen Denkansatzes auch argumentiert, wie sich derartige Inflationsszenarien wirtschaftspolitisch abwenden ließen. Denn eins ist genauso sicher: die Relation zwischen Naturkatastrophen und Inflation besteht seit je. Umso mehr, falls Inflationserscheinungen – wie heutzutage – nur zum Teil erkannt werden.

## Abstract (en)

Although COVID-19 pandemic has so far been assumed to have recessive-deflationary consequences due to the dramatic deterioration of economic forecasts worldwide, inflation risks cannot be excluded. What effects might result from the combination of billion-high liquidity injections and high decline in production levels as well as strict (and costly) security and protection requirements? And why should the term “inflation” be separated from “rising costs of living” from a macroeconomic perspective? By means of a logical-analytical approach, the following article also describes how in terms of economic policies such inflationary scenarios might be prevented. One thing is certain too: the relationship between natural disasters and inflation has always existed. Even more, if inflationary effects are—as today—only partially recognized.

## Einleitung: COVID-19-Pandemie und Geldpolitik.

Als das Coronavirus SARS-CoV‑2 nach Ausbruch in China erstmals im Januar 2020 der großen Öffentlichkeit bekannt geworden, zunächst seitens lokaler Behörden beschwichtigt worden („The pneumonia outbreak in Wuhan, will not evolve into a massive outbreak similar in scale to the SARS outbreak 17 years ago“ [National Health Commission of the People’s Republic of China 2020]) und bereits am 11. März 2020 von der Weltgesundheitsorganisation mit dramatischen Tönen zur „Pandemie“ heraufgestuft worden ist („We have never before seen a pandemic sparked by a coronavirus. This is the first pandemic caused by a coronavirus. And we have never before seen a pandemic that can be controlled, at the same time“ [Weltgesundheitsorganisation 2021]), hat man kaum Zeit gehabt, über sich daraus „binomialartig“ ergebende Wirtschaftseffekte nachzudenken, die im folgenden Beitrag aber thematisiert werden sollen. Weltweite Reisebeschränkungen, Geschäftssperrungen, streng einzuhaltende sowie beispiellose Sicherheits- und Schutzauflagen haben binnen weniger Monate zur dramatischen Verschlechterung aller noch geltenden Wirtschaftsprognosen geführt, was den diffusen Eingriff vonseiten Notenbanken entweder durch Liquiditätszuschüsse oder Leitzinssenkungen (z. B. in den USA um hundert Basispunkte von 1,25 auf 0,25 %) mit sich gezogen hat. Wie Zinssätze seitdem in vielen Ländern der Welt angepasst worden sind, soll Tab. 1 zeigen.

**Table 1:** 

Land	Notenbank	Zinssatz (%)	Datum und Basispunkte (BPS) der letzten Zinsänderung	
Australien	Reserve Bank of Australia	0,10	3. November 2020	−15 BPS
Brasilien	Banco Central do Brasil	2,00	5. August 2020	−25 BPS
China	People’s Bank of China	4,35	23. Oktober 2015	−25 BPS
Eurozone	European Central Bank	0,00	10. März 2016	−5 BPS
Indien	Reserve Bank of India	4,00	22. Mai 2020	−40 BPS
Japan	Nippon Ginkō	−0,10	29. Januar 2016	−20 BPS
Kanada	Bank of Canada	0,25	27. März 2020	−50 BPS
Neuseeland	Reserve Bank of New Zealand	0,25	15. März 2020	−75 BPS
Russland	Bank Rossii	4,25	24. Juli 2020	−25 BPS
Schweiz	Schweizerische Nationalbank	−0,75	15. Januar 2015	−50 BPS
Vereinigte Staaten von Amerika	Federal Reserve	0,00–0,25	15. März 2020	−100 BPS
Vereinigtes Königreich	Bank of England	0,10	19. März 2020	−15 BPS

Wenn die Januarausgabe des *World Economic Outlook* (WEO) das globale Wirtschaftswachstum für das Jahr 2020 noch auf +3,3 und das deutsche auf +1,1 % (Internationaler Währungsfonds 2020b) schätzte, wurde es bereits in der Oktoberausgabe auf jeweils −4,4 und −6,0 % (Internationaler Währungsfonds 2020c) gesenkt, um in der Juniausgabe nochmals auf jeweils −4,9 und −7,8 % (Internationaler Währungsfonds 2020b) heruntergeschraubt zu werden. Dass alle Länder weltweit von einem regelrechten Einbruch der Wirtschaftswachstumsprognosen heimgesucht worden sind, legt Tab. 2 deutlich offen. In diesem Zusammenhang haben etliche Notenbank der Schwere der Notlage entsprechend Stellung genommen: die Europäische Zentralbank (2020b) hat beispielsweise schon am 2. März 2020 verkündet, dass „the ECB is closely monitoring developments and their implications for the economy, medium-term inflation and the transmission of our monetary policy“, was wiederum (aufgrund der statutarischen Notwendigkeit, jede Entscheidung nur auf die Preisstabilität zurückzuführen) auf die Beschränktheit europäischer Geldpolitik – wenn mit jener der Federal Reserve verglichen – hinweist.

**Table 2:** 

	WEO (Januar 2020)		WEO (April 2020)		WEO (Oktober 2020)		Veränderung (Januar–Oktober 2020)	
	2020	2021	2020	2021	2020	2021	2020	2021
*Welt*	**+3,3**	**+3,4**	**−3,0**	**+5,8**	**−4,4**	**+5,2**	**−7,7**	**+1,8**
*Fortgeschrittene Länder*	**+1,6**	**+1,6**	**−6,1**	**+4,5**	**−5,8**	**+3,9**	**−7,4**	**+2,3**
Vereinigte Staaten von Amerika	+2,0	+1,7	−5,9	+4,7	−4,3	+3,1	−6,3	+1,4
*Eurozone*	**+1,3**	**+1,4**	**−7,5**	**+4,7**	**−8,3**	**+5,2**	**−9,6**	**+3,8**
Deutschland	+1,1	+1,4	−7,0	+5,2	−6,0	+4,2	−7,1	+2,8
Italien	+0,5	+0,7	−9,1	+4,8	−10,6	+5,2	−11,1	+4,5
Spanien	+1,6	+1,6	−8,0	+4,3	−12,8	+7,2	−14,4	+5,6
Vereinigtes Königreich	+1,4	+1,5	−6,5	+4,0	−9,8	+5,9	−11,2	+4,4
*Schwellen- und Entwicklungsländer*	**+1,4**	**+4,6**	**−1,0**	**+6,6**	**−3,3**	**+6,0**	**−4,7**	**+1,4**

Die amerikanische Notenbank hat diesbezüglich dank ihrem doppelseitigen Mandat aus Beibehaltung von Preisstabilität und Wirtschaftsstabilisierung einen Tag später hinzubemerkt, wie „the coronavirus poses evolving risks to economic activity. In light of these risks and in support of achieving its maximum employment and price stability goals, the Federal Open Market Committee decided today to lower the target range for the federal funds rate by 1/2 percentage point, to 1 to 1 1/4 %“ (Board of Governors of the Federal Reserve System 2020). Weitere präliminäre Statements reichen von der Bank of England (2020) – am 3. März 2020 hat sie verlautet: „[t]he Bank of England’s role is to help UK businesses and households manage through an economic shock that could prove large but will ultimately be temporary“ – bis hin zur Bank of Japan (2020), die mit besonderer Ausdruckskraft bereits am 2. März 2020 auf zu ergreifende geldpolitische Maßnahmen hinwies („provide ample liquidity and ensure stability in financial markets through appropriate market operations and asset purchases“). Es würde sicherlich den Rahmen der Abhandlung sprengen, die vielen COVID-19-bezogenen geldpolitischen Maßnahmen weltweit Revue zu passieren. Als Beispiel sei im Folgenden nur die Vorgehensweise der Europäischen Zentralbank (2020a) genannt: „[t]he €1,350 billion pandemic emergency purchase programme (PEPP) aims to lower borrowing costs and increase lending in the euro area. […] We have kept our interest rates at historically low levels […]. We have increased the amount of money that banks can borrow from us and made it easier for them to borrow specifically to make loans to those hardest-hit by the spread of the virus, including small and medium-sized firms. […] We are being temporarily less strict about the amount of funds, or ‚capital‘, that banks are required to hold as a buffer for difficult times. […] We have recently reactivated swap lines and enhanced existing swap lines with central banks across the globe in response to the current difficult situation“. Flankiert werden Notenbanken zurzeit zudem von den jeweiligen Regierungen, die zur Bekämpfung der COVID-19-Wirtschaftsfolgen großen Gebrauch fiskalpolitischer Maßnahmen machen („The goal oft he fiscal policy actions is to buffer the short-term impact of the shock. Governments have taken a wide array of measures to support individuals and firms. Central banks and financial regulators have complemented these actions with policies that have eased financial conditions and enabled the continued flow of credit to the real economy“ [Alberola et al. 2020]).

Solche Wirtschaftsmaßnahmen, mit denen einerseits Liquidität geschaffen und andererseits Staatsverschuldung genährt wird, haben allerdings aus dem Blickwinkel eines weiteren, geanuso außerordentlichen Ereignisses der jüngsten Wirtschaftsgeschichte betrachtet zu werden. Der Bezug ist wieder einmal auf die globale Finanz- und Wirtschaftskrise ab 2007, die schon damals aufgrund des Eingriffs seitens der Notenbanken für eine regelrechte Überflutung der Banken- und Finanzsysteme weltweit gesorgt hat („The ECB, together with the national central banks of countries in the euro area (the Eurosystem) has been lending unlimited amounts of money to banks in response to the financial crisis. In addition, it has been buying bonds from market participants. As a result, there is more money – or liquidity – in the banking system as a whole than is strictly needed. This is called excess liquidity“ [Europäische Zentralbank 2020c]). Nur ein Teil solcher exzessiven (d. h. vom globalen Realprodukt losgelösten) Liquidität ist aber absorbiert bzw. sterilisiert worden. Anders formuliert: auf bereits inflationäre (d. h. aus dem Nichts geschaffene) Liquidität soll jetzt noch weitere hinzukommen. Ob das „gut“ gehen wird?

## COVID-19-Maßnahmen als deflationär oder inflationär? erste Einschätzung.

Angesichts immer bedrückender Wirtschaftsszenarien, die von einer sich dramatisch entwickelnden Gesundheitslage begleitet worden sind – am 2. Februar 2021 gab es laut Weltgesundheitsorganisation (2021) 102.817.575 bestätigte Infizierungs- und 2.227.420 Todesfälle weltweit–, hat man sich kaum vorstellen können, dass Inflationsrisiken (vielleicht allzu bald) eine wichtige Rolle spielen können. Die meisten Ökonomen und Analysten gehen nämlich eher von Deflationserscheinungen aus, die ihrerseits das logischste Resultat der sich anbahnenden Rezession auf globaler Ebene wären („COVID-19 to plunge global economy into worst recession since World War II“ [Weltbank 2020a]). Solche Rückschlüsse sind zum jetzigen Zeitpunkt natürlich alles Andere als unplausibel. Wenn anfangs aber nur von „globaler Rezession“ und dementsprechend Deflationsszenarien aufgrund der Entwicklung von Maßwerten wie Rohöl (WTI), das am 28. Februar 2020 44,76 US-Dollar und am 30. April 2020 schon 18,84 US-Dollar preiste, um allerdings am 12. August 2020 bereits bei 42,23 US-Dollar zu liegen (finanzen.net 2020) die Rede gewesen ist, sind seit Mitte April 2020 erste (zögerliche) Warnrufe lauter geworden, nach denen es alsbald zu Inflationsschüben kommen könnte. Wie von Ralph Solveen in einem Interview mit Angelina Märkl (2020) geschildert, „[a]uf den ersten Blick erscheint die Sache sehr einfach: In den meisten Rezessionen der Vergangenheit ist die Teuerungsrate gefallen. Deshalb erwarten viele auch aktuell […] einen geringeren Anstieg oder gar einen Rückgang der Verbraucherpreise. Allerdings ist die aktuelle Rezession keine ‚normale‘ Rezession, Denn normalerweise kommt es zu einer Rezession, weil die Nachfrage einbricht. Der dann entstehende Angebotsüberschuss lässt die Preise fallen. Diesmal ist es anders: Auslöser für den Einbruch war der Lockdown, wegen dem das Angebot deutlich stärker gefallen ist als die Nachfrage. […] Aber es hatte zur Folge, dass die Sparquote der Privathaushalte kräftig gestiegen ist, auch weil die Einkommensausfälle durch Kurzarbeit oder Arbeitslosigkeit zu einem beträchtlichen Teil durch den Staat ausgeglichen wurden. Wäre die Pandemie morgen vorbei und jeder würde mit seinem Gesparten in die Läden stürmen, hätten wir auf einmal einen großen Nachfrageüberschuss. Gerade vor dem Hintergrund der Umsätzausfälle in den letzten Wochen würden dann sicherlich viele Anbieter ihre Preise anheben“. Egal, ob hochschnellende Nachfrage nach (gradueller) Aufhebung mit der COVID-19-Pandemie einhergegangenen Einschränkungen, besonders lockere Geldpolitik vonseiten der Notenbanken oder fiskalpolitische Wirtschaftsmaßnahmen: das Ergebnis in Sachen Endpreisen wäre das gleiche.

Obwohl die Wirtschaftswissenschaft zurzeit also mehrheitlich eher von deflationären Auswirkungen ausgeht, haben sich Stimmen, die doch vor möglichen Inflationseffekten warnen, gemehrt („We are unlikely to see much inflation in the near future. We might even see deflation, due to low aggregate demand. But inflationary pressures are likely to emerge after the engines of the global economy rev up again“ [Pastor 2020]). Eurostat (2020a, 2020b, 2020c) hat noch am 30. April 2020 den Rückgang jährlicher Inflationsrate im Euroraum von +0,7 auf +0,4 % gemeldet, die am 29. Mai 2020 nochmals nach unten auf +0,1 % korrigiert worden ist, um sie am 31. Juli 2020 erneut auf +0,4 % zu schätzen. Es herrscht also große Unsicherheit, aber Befürchtungen mittelfristiger Preisanstiege (die bislang vielleicht weniger offensichtlich gewesen sind) sind weniger abwegig geworden („Central banks […] will be under enormous political pressure to maintain supportive money printing at least until the economy has unambiguously returned to health. Having crossed the Rubicon into direct financing of budget deficits, that’s almost bound to be inflationary“ [Warner 2020]). Wirtschaftswissenschaftler „also learned how difficult it is to prevent those monetary diseases (inflation and deflation) which destroy this stability“ (Röpke 2008).

Allerdings hat man präzise vorzugehen, um Inflationsrisiken entsprechend einzustufen und adäquate wirtschaftspolitische Maßnahmen zu ergreifen. Unerlässliche (obgleich genauso unbeachtete) Voraussetzung ist, zwischen „Inflation“ (d. h. einem Preisanstieg, der Liquidität im Überschuss zum Realprodukt zuzuschreiben ist) und „höheren Lebenshaltungskosten“ (d. h. nicht inflationärem Preiswachstum) zu unterscheiden. Alvaro Cencini (2002) schildert diese definitorische Notwendigkeit wie folgt: „while inflation implies a loss of purchasing power for a given country’s money relative to current output, an increase in the cost of living can have negative repercussions for one or more groups of residents without necessarily modifying the relationship between national money and national output (the relationship that defines the purchasing power of national money)“. Die Frage, anhand der zwischen „inflationärem“ und „nichtinflationärem“ Preisanstieg unterschieden werden kann, lautet daher, ob das Verhältnis zwischen Produkt- und Geldmenge (das wiederum die Kaufkraft bestimmt und im Idealfall eins zu eins sein sollte) mutiert sei. Als Milton Friedman (1992) behauptete, dass „inflation is always and everywhere a monetary phenomenon […]. Inflation occurs when the quantity of money rises appreciably more rapidly than output, and more rapid the rise in the quantity of money per unit of output, the greater the rate of inflation“, hatte er zweifelsohne Recht. Dass eine derart wichtige Lehre trotz Friedmans eindeutiger Zugehörigkeit zum wissenschaftlichen Mainstream vernachlässigt wird, ist vielleicht umso denkwürdiger.

Allzu leicht vergessen wird also auch, dass nicht jeder Preisanstieg inflationären Ursprungs sein muss. Beispielsweise ließe sich die *spontane* (d. h. *freiwillige*) *Entscheidung* selbst eines Industriesektors, die Endpreise dessen Güter und Dienstleistungen anzuheben, um Investitionen in Innovation und Technik zu fördern, keineswegs *tout court* Inflation zuschreiben, obgleich letztendlich ein Preisanstieg von den gängigen Indizes verzeichnet werden würde. Das Verhältnis zwischen Geld- und Produktmenge wäre nämlich *ceteris paribus* das gleiche wie vor den Preiserhöhungen. Zusammengefasst: Preisanstieg ja, Inflation nein. Dass letztere hingegen immer ein *erzwungenes *(d. h. *unfreiwilliges*) Preiswachstum auslöst, rührt hingegen davon her, dass bei überschüssiger Liquidität (z. B. 120 Geldeinheiten) das Realprodukt (z. B. 100 Produkteinheiten) unter Wert verkauft werden und mit einem subproportionalen Aufwand an Geldeinheiten $\left(\frac{100}{120}=83,33\% \right)$ aufgebraucht werden würde. Verkaufspreise *müssen* daher angehoben werden, um die zu geringe Anzahl an Geldeinheiten, die zum potenziellen Erwerb des gesamten Realprodukts nötig wären, zu kompensieren. In diesem Fall würde es sich aber tatsächlich um einen inflationären Preisanstieg handeln. Das zweite Beispiel hat also nichts mit dem ersten zu tun. Dem könnte wie häufig auch entgegnet werden, dass Konsumausgaben bei Vorliegen obiger Bedingungen (d. h. ansteigenden Preisen) allmählich geringer ausfallen würden, was den Aufwärtstendenz abbremsen würde. Im Falle eines derartigen Einwands würde man aber Mikro- mit Makroökonomik (d. h. *verhaltensbedingte* mit *-losgelöster* Analyse) vermischen. Selbst wenn weniger konsumiert und mehr gespart werden sollte, wären solche Geldressourcen *zwangsläufig* anderweitig angelegt: sogar falls sie (nicht angelegt) auf Bankkonten verbleiben würden, würden sie trotzdem zum Tagesgeschäft von Banken beitragen und den Geldkreislauf antreiben. Wie Alvaro Cencini (2002) erneut erklärt, „[t]o imagine that a person can decrease the sum of bank deposits without spending (and, therefore, without exerting an equivalent demand), amounts to assuming, erroneously, that money identifies itself with the material supports used to represent it. A person can hide bank notes in his garden or under his mattress, of course, but this does not not mean that, by doing so, he destroys the book-keeping entries corresponding to the bank notes withdrawn from circulation and recorded by banks at the moment of their emission. Bank notes can be hoarded, money cannot. To hoard bank notes amounts to taking claims on bank deposits out of circulation, but not the bank deposits themselves. Since demand is determined on the basis of the income available to finance it, it immediately followst hat it is fundamentally independent from the behaviour of those who deposit their income within the banking system“. Inflationsdruck wäre – selbst wenn nicht am Konsumgütermarkt – also genauso präsent.

Zwischen „Lebenskostensteigerung“ und „Inflation“ – egal welche spezifischen Realwerte betroffen sein sollten („variations in oil prices or weather conditions may raise prices for individual items, but they cannot raise prices for all goods, as inflation does. A general rise in the prices of all economic goods is possible only if there is […] an unwanted increase in the quantity of money“ [De Marcos 1961]) – liegt ein wesentlicher sowie vernachlässigter Unterschied vor. Die Analyse von „Inflation“ und „Lebenshaltungsmehrkosten“ kann auch anhand eines weiteren Kriteriums erfolgen, wie folgendes Beispiel offenlegt: „[t]here are increases in the price index which can be referred to as causes of an increase in the cost of living but which have nothing to do with inflation, that is, with a pathological reduction in money’s purchasing power. On the contrary, there are cases in which the presence of inflation is not revealed by a variation in the price index“ (Cencini 2005). Nur von Inflationsdruck ausgehende Steigerungen des Preisniveaus sind als „pathologisch“ bzw. „makroökonomischen“ oder „strukturellen“ Ursprungs betrachtet worden, während entscheidungsbedingte Preissteigerungen – um sich davon beispielsweise höhere Erträge und Gewinne zu erhoffen oder weitere Unternehmungen zu finanzieren – mikroökonomischer (d. h. nicht „struktureller“) Natur sind. Tab. 3 soll anhand numerischer Beispiele die obigen Ausführungen (d. h. die Kriterien, um zwischen „Lebenskostensteigerung“ und „Inflation“ zu unterscheiden) zusammenfassen.

**Table 3:** 

Beispiel	Produktmenge	Geldmenge	Verhältnis zwischen Produkt- und Geldmenge	Wirtschaftsphänomen	Bemerkungen
*1*	100 Einheiten	100 Einheiten	$\frac{100}{100}=1$	Weder Inflation noch Deflation	Preise können immerhin aus entscheidungsbedingten (d. h. nichtinflationären Gründen) variieren. Das können sie theoretisch auch in Beispiel 2. und 3
*2*	100 Einheiten	80 Einheiten	$\frac{100}{80}=1,25$	Deflation	Preise müssen sinken, um den potenziellen Erwerb des gesamten Realprodukts (100) bei zu knapper Liquidität (80) zu ermöglichen
*3*	100 Einheiten	120 Einheiten	$\frac{100}{120}=0,83$	Inflation	Preise müssen steigen, damit das gesamte Realprodukt (100) nicht mittels zu hoher Liquidität (120) potenziell zu „leicht“ erworben (und aufgebraucht) werden kann

Obwohl die Coronaviruskrise mit ihrem dramatischen Einbruch globaler Wirtschaftsprognosen als Folge auf Anhieb zu einem entsprechenden Deflationsrisiko tendieren ließe, könnte der Schein allzu bald trügen. Zum einen aufgrund der ergriffenen Wirtschaftsmaßnahmen, die weitere Liquidität (bei stark rückläufigem Realprodukt) in das globale Wirtschaftssystem einpumpen werden, zum anderen weil unter „Inflation“ zumeist ein Anstieg bei den Verbraucherpreisen verstanden wird. Die ganze Geschichte ist dies aber allemal nicht.

## Auswirkungen von COVID-19-Wirtschaftsmaßnahmen auf unterschiedliche Inflationskonzepte

**Figure 1:**
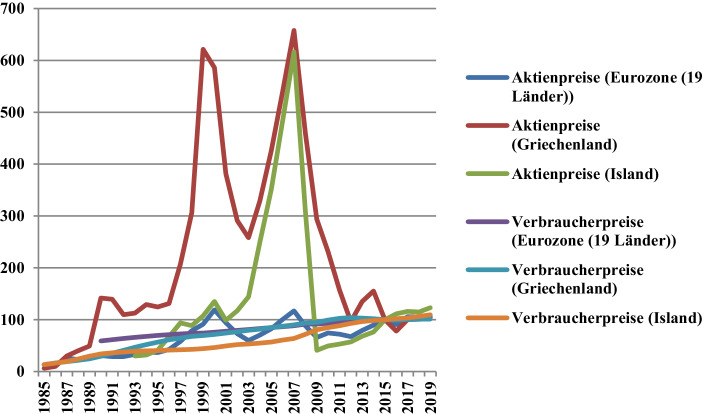

Dass Inflationsschübe sich in postindustriellen Wirtschaftssystemen immer weniger auf Konsumgütermärkte ausschlagen (auf denen ein immer geringerer Anteil individuellen Einkommens ausgegeben wird), sie sich dafür immer mehr auf ertragsstärkere Sektoren wie Immobilien- oder Finanzmärkte auswirken und für so genannte *asset price inflation* sorgen, kann wohl kaum ein Geheimnis sein. Nichtsdestotrotz wird diese Tatsache von üblichen Verbraucherpreisindizes nicht im Geringsten berücksichtigt. Es sei beispielsweise darauf hingewiesen, wie laut Eurostat (2020d) „[h]ouse prices, as measured by the House Price Index, rose by 5,0 % in the euro area in the first quarter of 2020 compared with the same quarter of the previous year. This is the highest annual increase since the second quarter of 2007. In the EU house prices rose by 5,5 % compared with the same quarter of the previous year“. Dass es sich bei Folgendem um ein zu „weites Feld“ (Fontane 1896) handelt, um es in wenigen Zeilen zu vertiefen, ist mehr als selbstverständlich. Allerdings reicht in diesem Zusammenhang ein weiteres, besonders prägnantes Beispiel aus der jüngsten globalen Finanzkrise aus: Griechenland und Island, die bekanntlich von massiver Spekulation und dominoartigen Insolvenzen geplagt worden sind, haben laut Organisation für wirtschaftliche Zusammenarbeit und Entwicklung (2020b) im Jahre 2007 bei ihren Aktienpreisen jeweils einen Höchstwert von 657,8 und 616,5 Punkten (wobei 2015 = 100) verzeichnet. Dass im selben Jahr der Durchschnittswert in der Eurozone bei 116,9 Punkten lag, weist erneut in aller Klarheit darauf hin, wie sich überschüssige (d. h. inflationäre) Liquidität im isländischen und griechischen Fallbeispiel vor allem am Aktienmarkt angesammelt und dort für „blasentypische“ Preisanstiegen gesorgt hat. Der Verbrauchpreisindex meldete in Griechenland hingegen nur einen Anstieg von +2,9 und in Island von +5,05 % (Organisation für wirtschaftliche Zusammenarbeit und Entwicklung 2020a). Wie der Kursanstieg im griechischen und isländischen Fall halsbrecherisch schnell (und spekulativ) gewesen ist, so ist es der bald darauf gefolgte Kurssturz gewesen, als die soeben genannte überschüssige (d. h. inflationäre) Liquidität abgeflossen oder -gezogen worden ist. Abb. 1 hebt diesen Zustand besonders deutlich hervor.

Als Schreibender ist man sich natürlich bewusst, dass Wirtschaftsdenker eher von deflationär-rezessiven Kräften ausgehen („The impact of the coronavirus on inflation is uncertain, as there are simultaneous supply and demand movements that can tilt the balance towards more inflation, disinflation, or even deflation. In the short term, despite measurement problems and the closure of markets, disinflation has dominated. In the medium term, several factors suggest that disinflationary pressures will continue to dominate“ [Leandro und Llorens i Jimeno 2020]). Allerdings ist wieder einmal entscheidend, was unter dem Begriff „Inflation“ verstanden wird. Es besteht beispielsweise kein Zweifel, dass Einbrüche an Finanzmärkten weltweit milliardenhohe Beträge an Liquidität vernichtet haben – „am Ende des Handelstages 18. März 2020 schloss der DAX bei 8441,4 Punkten“ (Statista 2020) –, aber nach Erreichen der 13.718,80-Punkte-Marke am 30. Dezember 2020 (Boerse.de 2021) waren sie bereits wettgemacht worden. Damit ist er also auf dem besten Weg, die von COVID-19 verursachten Aktienmarktverluste wettzumachen. Zugleich weiß man bereits, dass Notenbanken und Staaten milliardenschwere Liquiditätsspritzen zugunsten der jeweiligen Nationalwirtschaften vornehmen werden. Diesbezüglich hat die US-Regierung schon verkündet, von „Helikoptergeld“ – Ironie des Schicksals: da wäre man erneut bei einem von Milton Friedman geprägten Begriff – Gebrauch machen zu wollen. Genauer gesagt „[h]elicopter money is not a new form of central bank money or a new way of creating it; it is just a different way of distributing it and pouring it into the economy, without going through the banks and financial markets. In the current phase, […], helicopter money should go to the States, to monetize public spending. Then, in a second phase, […], helicopter money could be paid to households and businesses to increase private spending without delay and without transmission failure“ (Couppey-Soubeyran 2020).

Zugleich haben massive weltweite Produktionseinschränkungen für einen dramatischen Rückgang bei der Herstellung von Gütern und Dienstleistungen gesorgt, was die bereits erwähnte Relation zwischen Realprodukt und Geldeinheiten zugunsten letzterer neigen lassen und den Überhang von Liquiditätsvolumina gegenüber Realwerten markieren könnte. Dass zugleich eine enge Verbindung zwischen exzessiver Liquidität, die ihrerseits zu übermäßiger Kreditvergabe oder „ungedeckter“ Finanzierung von Konsumausgaben beiträgt, und privater sowie öffentlicher Überschuldung besteht, wird vom Schreibenden in einem Online-first-Artikel für *Economic notes* mit dem Titel *The fourfold relation between the essence of money, inflation, bubbles and debt – A theoretical macrofounded analysis* erläutert. Der Überhang von Finanz- gegenüber Realwerten wird von der Wirtschaftsliteratur schon seit Jahren dokumentiert: „[a]ll expenditure in a period of time has to be funded either by current income or by additional credit and debt taken up during that time, or, individually, by liquidation of assets. Financial and earned incomes add up to 100 %. […] A build-up of monetary and financial assets disproportionate to GDP thus creates a distributional bias in favor of financial income, resulting in a reduced share of earned income. […] The more financial assets grow GDP-disproportionately, the bigger the share that goes into unproductive non-GDP transactions which nonetheless demand to be serviced by the flow of actual income and additional debt“ (Huber 2016).

**Figure 2:**
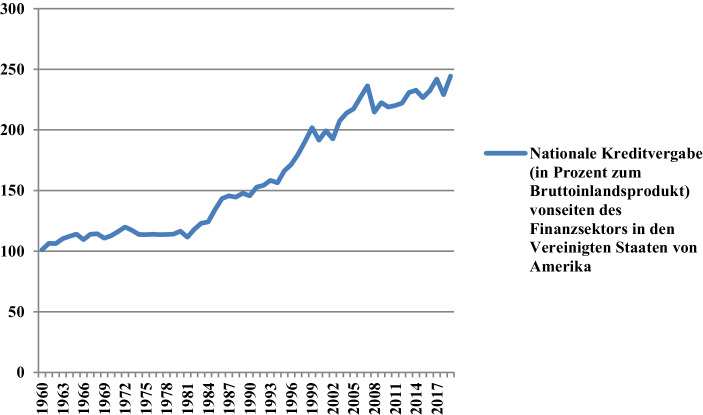

**Figure 3:**
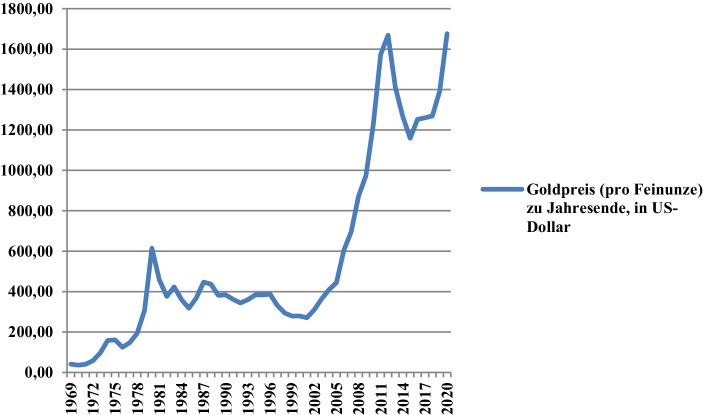

Angesichts der Tatsache, dass einerseits die nationale Kreditvergabe des Finanzsektors von Ländern wie den Vereinigten Staaten von Amerika im Vergleich zum Bruttoinlandsprodukt schon seit Jahrzehnten die 100-Prozent-Marke überschreitet (Abb. 2) sowie massive Liquiditätsspritzen hinzukommen werden und das globale Realprodukt zurzeit andererseits einen dramatischen Rückgang – es sei gesagt: potenziell unendlich dehnbaren, solange Begriffe wie „Quarantäne“ oder „Lockdown“ statt „Massenimpfung“ die Schlagzeilen beherrschen – verzeichnet, ist der Überhang der Geld- zur Produktmenge mehr als offensichtlich. Ob die überschüssige Liquidität am Konsumgütermarkt investiert werden (und dort Preissteigerungen nähren) wird, ist jedoch mehr als fraglich. Zumindest ein Teil davon wird es, aber der Großteil wird weiterhin an den lukrativsten Märkten angelegt werden. Erst vor Kurzem ist erneut gemeldet worden, dass selbst im 2. Quartal 2020 „[d]er Index für Eigentumswohnungen mit einem Zuwachs von 2,6 % gegenüber dem letzten Quartal am stärksten angestiegen [ist]. […] Auch die Mietpreise ziehen weiter an, mit einem Wachstum von 1,1 % gegenüber dem letzten Quartal zum wiederholten erheblich schwächer“ (empirica 2020). Ob ein solcher Trend verwundern soll? Wohl kaum, denn schließlich lässt sich die Geschichte immer weiter konjugieren. Ein weiteres Beispiel für potenziell inflationäre Aufblähung bekannter Wertmaßstäbe liefert zurzeit das Epitom von Reichtum schlechthin, nämlich das Gold. Dass das gelbe Edelmetall schon immer hochbegehrt ist, weiß man. Was eher in den Hintergrund treten mag, ist, dass selbst Gold noch vor Kurzem weitaus weniger preiste, wie Abb. 3 in aller Deutlichkeit zeigt.

Als in der zweiten Nachkriegszeit der US-Dollar das Sterling Pfund als Zahlungsmittel bei kommerziellen und finanziellen Transaktionen weltweit ersetzt hat, ist Gold vor allem als Garantie für die Konvertibilität des US-Dollar betrachtet worden. Damals preiste eine Feinunze (31,10 g) allerdings noch 35 US-Dollar. Am 15. August 1971 beschloss US-Präsident Richard Nixon die Goldkonvertibilität der amerikanischen Währung aufzuheben, zumal die in Fort Knox gelagerten Goldreserven immer knapper wurden. Als vom 6. bis 7. Januar 1976 in Kingston (Jamaika) der Internationale Währungsfonds zudem bekannt gab, einen Teil der internationalen Goldreserven verkaufen zu wollen, wäre es letztendlich naheliegend gewesen, zu meinen, dass Gold schlussendlich zu jenem „mittelalterlichen Relikt“ (*barbarous relic*, wie Ökonomen oft sagen) verkommen wäre. Schließlich war es (und ist es immer noch) mit einem chronischen Entstehungsfehler behaftet: seiner Knappheit in stark wachsenden Wirtschaftssystemen, die ebenso rapide steigender Geldbestände bedürfen. Die Geschichte lehrt uns dennoch das Gegenteil und – siehe da – Gold wird je heftiger die Wirtschaftskrise zum umso gesuchten Rettungsanker. Was in diesem Kontext aber noch interessanter ist, ist, dass man seit Langem – allerspätestens seit der Vorphase der globalen Finanz- und Wirtschaftskrise – von jenen 35 US-Dollar pro Feinunze weit entfernt ist. Was kann aber passiert sein, dass der Goldpreis seit kurz vor dem Ausbruch der Subprime-Krise immer haarsträubendere Höchstwerte erreicht? Hat die postindustrielle Gesellschaft nun das Gold als wiederentdeckt? Wohl kaum. Viel eher lässt sich ein solch historisch abdriftender Goldkurs damit erklären, dass ein Teil der weltweit herumirrenden Überschussliquidität (die also inflationär ist) stabil am Edelmetallmarkt angelegt ist. Ob dies auch im Zuge der COVID-19-Pandemie so sein wird, ist fraglich, zumal inflationäre Liquidität – je nach Investitions- oder Desinvestitionsentscheidungen – ihr „Objekt der Begierde“ genauso abrupt wechseln kann. Der gemeinsame Nenner bleibt dennoch das Stichwort des vorliegenden Beitrags, nämlich „Inflation“.

## Weitere Gründe für COVID-19-verbundenen Preiserhöhungen

Parallel zu derart von überschüssiger Liquidität angetriebener Inflation ist auch kaum auszuschließen, dass Unternehmen selbst, die zunächst wochenlang auf Lockerungen der Produktionseinschränkungen haben ausharren müssen und später den Betrieb nur langsam wiederaufnehmen haben können, Endpreise schon aufgrund kostenträchtiger Sicherheits- und Schutzmaßnahmen anheben könnten. Das Statistische Bundesamt (2020) weist jedenfalls bereits nach, wie „Arbeitskosten je geleistete Arbeitsstunde im Zeitraum vom 1. Quartal 2019 zum 1. Quartal 2020 kalenderbereinigt um 4,3 % gestiegen [sind]. Dies ist die höchste Veränderung, die der Arbeitskostenindex jemals ausgewiesen hat. […] [Dieser Anstieg] lässt sich vor allem auf einen Rückgang der geleisteten Arbeitsstunden zurückführen“. Es sei hier noch ein weiteres, vielleicht noch konkreteres Beispiel genannt: laut einer Umfrage, an der 1067 Salonunternehmen beteiligt gewesen sind, hätten sich 55,7 % der Befragten für eine Preiserhöhung und 30,1 % für eine Kostenweitergabe durch eine Hygieneabgabe entschieden. Nur 11 % würden von einer Preiserhöhung absehen, während weitere 3,3 % noch abwarten wollten (imsalon.de 2020). Es ist jedenfalls mehr als plausibel, dass schon wegen der ergriffenen Lockdownmaßnahmen zuerst und strengen Wiedereröffnungsauflagen danach Unternehmen die von ihnen getragenen Mehrkosten an die Kundschaft weitergeben werden. Aus diesem Grund könnte die Coronakrise schon wegen ihrer wesentlichen Unterschiede zur globalen Finanzkrise ab 2007 (die schlussendlich endogen zum Wirtschaftssystem gewesen ist) zu einer kaum zu unterschätzenden Schmälerung der Individualkaufkraft beitragen.

Dass Naturkatastrophen – Pandemien zählen selbstverständlich zu dieser Kategorie – schon lange in enger Relation zu Inflationserscheinungen (als Folgeeffekt) stehen, lässt sich analytisch nachvollziehen sowie empirisch belegen. In dieser Hinsicht hat Miles Parker (2006) geschlussforgert, dass „the impact for less developed countries is more marked, with significant effects on headline inflation persisting even three years post-disaster. That said, there is a significant impact in high income countries from severe disasters“. Bereits am 29. April 2020 hat das Australian Bureau of Statistics (2020) den höchsten Inflationsstand seit September 2014 gemeldet, der seinerseits Dürre und Waldbränden (d. h. Naturkatastrophen) zugeschrieben worden ist. Wenn man dem hinzubemerkt, dass es sich um „typische“ australische Naturkatastrophen handelt (die einigermaßen schon „eingepreist“ sind), kann man sich den umso zerrüttenden Effekt aus weitaus weniger „normalen“ (exogenen) Ereignissen wie Pandemien nur allzu gut vorstellen.

Es hat jedoch in Frage gestellt zu werden, ob Verbraucherpreisindizes solche Preisbewegungen (die selbst nichtinflationärer Natur sein können) auch (gesamtheitlich) verzeichnen werden. Neben methodologischen Vorgehensweisen, die für eine gewisse Unterschätzung deklarierter Preisanstiege sorgen können („[b]eginning in the 1990s, many US policymakers […] began to grow increasingly concerned about potential upside biases in CPI. […] The call to reduce published inflation figures was very good news politically […] .[…] the implemented methodological tweaks have helped to significantly reduce official inflation numbers over the past 30 years“ [Hochstein 2020]), existiert auch das Wirtschaftsphänomen der „Shrinkflation“. Damit wird vor allem die Reduzierung – man vergesse aber nicht: auch die Qualitätsschmälerung – der Verkaufsmenge bei gleich bleibendem Preis bezeichnet, wie das Office for National Statistics (2019) bei verschiedenen Produkten zwischen 2015 und 2017 erörtert hat: diesbezüglich „[t]he majority of products experiencing size changes were food products and in 2016, we estimated that between 1 % and 2,1 % of food products in our sample shrank in size, while between 0,3 % and 0,7 % got bigger“.

Wenn hier schon Schluss wäre, könnte man sich mit diesem Status quo getrost abfinden. Natürlich müsste man sich darüber im Klaren sein, dass solche Ergebnisse nur bei genauen (und entsprechend mühsamen) statistischen Nachforschungen zu erhalten wären. Anders formuliert: statistische Ämter – Verbraucherpreisindizes allein umso weniger – würden „im Normalfall“ wohl kaum jedwede „Shrinkflation“ registrieren. Was wäre aber, wenn die Qualität (d. h. nicht unbedingt die Größe) des erfassten Gutes schlechter wäre, um Produktionskosten zu drücken? Auch in diesem Fall würde man kaum von „Shrinkflation“ ausgehen, obwohl sie bei der Kundschaft für ein schlechteres Produkt zu einem gleichen Preis sorgen würde. Doch wäre sie da. Ein derartiger listiger Mechanismus ist jedenfalls schnell umsetzbar: ein Service weniger im gleichen Reisepaket hier, eine Rezepturanpassung bei günstigeren Zutaten da und schon würde es für jegliche Statistiken unmöglich werden, davon Wind zu bekommen.

Es wäre daher unpassend, zu glauben, dass infolge von COVID-19 Verkaufspreise (aufgrund der sich anbahnenden globalen Rezession) schrumpfen würden. Man hat bereits konstatiert, wie die Weltwirtschaft bislang eher von deflationären Kräften ausgeht („for AES, the output collapse due to the Covid crisis is associated mainly with greater downside risks to inflation in the near term. For some EMEs, the exchange rate depreciation appears to lead to a prominent increase in upside risks to inflation. Moreover, tighter financial conditions seemingly contribute to both downside and upside inflation risks“ [Banerjee et al. 2020]), obwohl sich auch skeptischere Stimmen mehren („Alles in allem ist das Risiko einer höheren Inflation für die nächsten Jahre weitaus größer als das Risiko einer Deflation. Der wesentliche Treiber is eine expansive Wirtschaftspolitik, die angesichts sehr hoher Schulden auf höhere Inflationsraten abzielen wird“ [Heise 2020]). Solche Analysen sollen jedenfalls damit ergänzt werden, dass – was von Statistikämtern als „Inflation“ bezeichnet werden würde – vor allem ein Preisanstieg bei den Verbrauchergütern und -dienstleistungen sein würde, der inflationärer (d. h. liquiditätsbedingter), aber auch nichtinflationärer (d. h. unternehmerischer) Natur sein könnte. Die Problemursache würde also weiterhin unentdeckt bleiben.

## Wirtschaftspolitische Implikationen: ein Schlusswort

Wie ließen sich aber die obigen Inflationsszenarien abwenden, wenn jetzige Wirtschaftseingriffe wie Liquiditätseingaben kaum beanstandet werden können? Zuallererst ist eine Erweiterung bzw. Vervollständigung des Inflationsbegriffs mehr denn je unerlässlich. Es genügt neben den bisherigen Beispielen ein letztes aus der Schweiz zu nennen, wo eine Krankenversicherungspflicht herrscht. Der Logik nach – falls man zumindest für einen Augenblick vom Begriff „Konsumgut“ abstrahieren würde, der gängigen Verbraucherpreisindizes obliegt – müsste ein derart relevanter, obligatorischer Ausgabeposten in der Berechnung der Inflationsrate inbegriffen sein. Die Rede ist hier gewiss nicht von geringfügigen Prämienbeträgen: die mittlere Prämie pro Versicherten betrug im Jahre 1996 nämlich 1539 Schweizer Franken, während sie im Jahre 2018 bereits bei 3735 Schweizer Franken (Bundesamt für Gesundheit 2020; Beretta 2020) lag. Aber genau eine derart naheliegende Einberechnung findet nicht statt. Anders formuliert: der Anstieg der mittleren Prämie pro Versicherten um 142,69 % zwischen 1996 und 2018 hat sich wieder einmal „unbemerkt“ vom entscheidungsrelevanten Inflationsindex zugetragen.

Nur mithilfe der obigen Anpassung ließen sich sowohl traditionelle Konsumgütermärkte (die in dieser Hinsicht bereits gedeckt sind) als auch all jene mit anderen Wirtschaftssektoren zusammenhängende Preisentwicklung erfassen, die sich in den letzten Dekaden vor allem in fortgeschrittenen Ländern infolge liquiditätsüppiger Wirtschaftsmaßnahmen allzu oft als regelrechte „Brutstätten“ systemischer Finanzkrisen erwiesen haben. Mit Bezug auf die globale Finanz- und Wirtschaftskrise ab 2007‑8 lässt sich zu Recht behaupten, dass „[t]he prevalent view is that the current credit crisis has its origin in the bust of the housing bubble. But what is missing from this view is that the finance of a bubble is only possible through a corresponding increase in credit – no credit, no bubble. Thus at the heart of the current woes lies the excessive liquidity that was put in place in the last ten years or so“ (Arestis und Karakitsos 2009).

Zudem hat sichergestellt zu werden, dass in der Zeit nach der Pandemie auf die jetzigen sowie folgenden Liquiditätsspritzen eine vorsichtige Drosselung der Geldzufuhr folgen wird, wobei letztere nicht zwangsläufig über Zinsanhebungen, die letztendlich die Realwirtschaft insgesamt belasten, erfolgen müsste. Eine solche Liquiditätssterilisierung könnte eher über die Straffung der Geldschöpfungskriterien im Interbankengeschäft verlaufen. Es sollte hierbei nämlich nicht vergessen werden, wie gerade das Interbankengeschäft über elektronische Geldschöpfung (zu Nullkosten also) für enorme, potenziell inflationäre Liquiditätsvolumina sorgt („Money is a spontaneous acknowledgement of debt issued by banks. […] Yet, its emission would be meaningless if the object of this debt was bound to remain money. In other words, the emission of money acquires all its significance only if it is associated with an economic transaction endowing it with a positive value. The idea that money can be issued as a positive amount of income irrespective of production is thus wrong. It would amount to claiming that banks can create a positive value out of thin air“ [Cencini und Rossi 2016]). Bereits im Jahre 2018 lagen die vom Finanzsektor vergebenen Inlandskredite laut Weltbank (2020b) bei 141,81 % im Vergleich zum Bruttoinlandsprodukt, wie Abb. 2 offenlegt. Der Zinssatz spielt bei derartigen Liquiditätsdynamiken nur bedingt eine Rolle, weil er lediglich die Kreditaufnahme (z. B. über Zinssenkungen) fördern oder von ihr (vgl. über Zinserhöhungen) entmutigen kann. Jegliche inflationäre Geldschöpfungspolitik vonseiten des Bankensystems wird damit kaum verhindert, zumal Bank- und Finanzinstitute im Falle einer weniger vorteilhaften Zinslage ihr Geschäft so anlegen können, damit keine Verluste eingefahren werden würden.

Was potenziell steigende Lebenshaltungskosten (d. h. die oben genannten „freiwilligen Preisanhebungen“ vonseiten Verkäufer) angehen würde, wären sie wie schon erwähnt nicht mit Inflation zu verwechseln, sondern lediglich auf die Absicht zurückzuführen, eingefahrene Ertragsverluste so effektiv wie möglich wettzumachen. Solch kompensatorische Zwecke der Preissteigerung mögen getrost auf die Brieftasche der Gesellschaft insgesamt schlagen, aber das Kriterium zur Erkennung von Inflation kann wohl kaum die Anzahl betroffener Individuen, sondern soll nur deren monetärer Ursprung sein. Nichtsdestotrotz sollte diesem Wirtschaftsphänomen genauso viel Aufmerksamkeit vonseiten Entscheidungsträger geschenkt werden, weil es das gesellschaftliche Lebensniveau ebenso schmälern und die Ungleichheitsschere weiten. Auch Geschäftsleute werden allerdings aus unternehmerischer Sicht nachvollziehen müssen, dass – wie die Coronakrise mit ihren Einschränkungen Geduld erfordert – man sich so viel Zeit auch nehmen werden muss, um Verluste zu kompensieren und Kundschaft nicht zu verjagen.

Naturkatastrophen und Preiseffekte weisen jedenfalls schon lange – auch nur aufgrund der Tatsache, dass sie stark schrumpfendes Angebot von Gütern und Dienstleistungen mit weniger markantem Nachfragerückgang kombinieren – eine doppelfädige Relation auf. Wenn zu diesem Thema eher wenig Literatur vorliegt, hängt es vor allem damit zusammen, wie Pandemien als (eher unwahrscheinliches) Worst-Case-Szenario empfunden werden, deren makroökonomische Folgen genauso häufig unterschätzt zu werden drohen. Ein Beispiel dafür soll im Folgenden geliefert werden: „we have estimated the costs of a pandemic using a macro-model for the EU-25. […] we end up with an estimate of the GDP loss ranging between 2 and 4 per cent. […] Our estimate of the macroeconomic cost of a pandemic in Europe is high, as we have investigated a rather severe medical scenario with a mortality rate higher than that of the Spanish influenza in Europe in 1918–1920. Still, such a pandemic does not have to spell economic disaster for Europe. The macroeconomic effects of a future pandemic as estimated here are roughly of the same size as those of a major recession“ (Jonung und Roeger 2006). Weitere COVID-19-bezogene Studien gehen hingegen von einem „annual output loss […] between 5 and 9 % of pre-Covid-19 estimates for the US, and between 4 and 4,5 % for the global economy. In worse scenarios, these costs could reach 11 % for the US and 8 % for the global economy“ (Boissay und Rungcharoenkitkul 2020).

Dass die letzten *World-Economic-Outlook*-Ausgaben sogar noch eines Besseren belehren – sprich: wirtschaftliche Schreckensszenarien bei Weitem nicht gebannt sind – , belegt Tab. 2 besonders gut. Während die Berechnung des Bruttoinlandsprodukts aber von einer gewissen Kohärenz geprägt ist, zumal sie den Gesamtwert aller Endprodukte in Gestalt von Gütern und Dienstleistungen (nach Abzug aller Vorleistungen) während eines Jahres innerhalb einer Volkswirtschaft erfolgt und alles nicht (offiziell) Entlohnte ausschließt, sind Angaben zu Preissteigerungen weitaus weniger konsistent. Ein vollständiger Überblick zu Teuerungstrends – auch im Zuge der COVID-19-Pandemie – wird jedenfalls vom Verbraucherpreisindex kaum gewährleistet. Es wäre dennoch ein gravierender Irrtum, davon auszugehen, dass Inflationserscheinungen, aber selbstnichtinflationäre Preisanstiege Wirtschaftssystemen inne liegend (und dementsprechend unanwendbar) wären. Bei aufmerksamer Geld- und Wirtschaftspolitik – aber nur unter solchen Umständen – wären sie es keineswegs.

## References

[CR1] Alberola, E., Arslan, Y., Cheng, G., & Moessner, R. (2020). The fiscal response to the Covid-19 crisis in advanced and emerging market economies. *BIS Bulletin No. 23*. http://www.bis.org/publ/bisbull23.pdf. Zugegriffen: 13. Aug. 2020.

[CR2] Arestis P, Karakitsos E, Arestis P, Mooslechner P, Wagner K (2009). Subprime mortgage market and current financial crisis. Housing market challenges in Europe and the United States. Any solutions available.

[CR3] Australian Bureau of Statistics (2020). CPI rose 0.3 per cent in the March 2020 quarter—Media release, 29 April 2020. http://www.abs.gov.au/ausstats/abs@.nsf/lookup/6401.0Media Release1March 2020. Zugegriffen: 13. Aug. 2020.

[CR4] Banerjee, R., Mehrotra, A., & Zampolli, F. (2020). Inflation at risk from Covid-19. *BIS Bulletin No. 28*. http://www.bis.org/publ/bisbull28.pdf. Zugegriffen: 13. Aug. 2020.

[CR5] Bank of England (2020). Governor statement to Treasury Select Committee, on behalf of the FPC, MPC and PRC—Published on 03 March 2020. http://www.bankofengland.co.uk/news/2020/march/governor-statement-to-tsc-on-behalf-of-the-fp-mp-and-pr-committees. Zugegriffen: 13. Aug. 2020.

[CR6] Bank of Japan (2020). Statement by the Governor—March 2. http://www.boj.or.jp/en/announcements/press/danwa/dan2003a.pdf. Zugegriffen: 13. Aug. 2020.

[CR8] Beretta E (2020). Premi della cassa malati: scongiurare ulteriori rialzi. Azione.

[CR9] Board of Governors of the Federal Reserve System (2020). Federal Reserve issues FOMC statement—March 03. http://www.federalreserve.gov/newsevents/pressreleases/monetary20200303a.htm. Zugegriffen: 13. Aug. 2020.

[CR11] Boissay, F., & Rungcharoenkitkul, P. (2020). Macroeconomic effects of Covid-19: an early review. http://www.bis.org/publ/bisbull07.pdf. Zugegriffen: 13. Aug. 2020.

[CR12] Bundesamt für Gesundheit (2020). T 1.01 Obligatorische Krankenpflegeversicherung ab 1996: wichtigste Indikatoren. http://www.bag.admin.ch/dam/bag/de/dokumente/kuv-aufsicht/stat/publications-aos/STAT%20KV%2018xls.zip.download.zip/_STAT%20KV%202018%20XLSX%20german%20and%20french%20v191107.zip. Zugegriffen: 13. Aug. 2020.

[CR14] Cencini A (2002). Monetary theory. National and international.

[CR13] Cencini A, Baranzini M, Cencini A (2005). Inflation and deflation. The two faces of the same reality. Inflation and unemployment: contributions to a new macroeconomic approach.

[CR15] Cencini A, Rossi S, Rochon L-P, Rossi S (2016). Inflation and unemployment. An introduction to macroeconomics. A heterodox approach to economic analysis.

[CR16] Couppey-Soubeyran, J. (2020). “Helicopter money” to combat economic depression in the wake of the Covid-19 crisis. http://www.veblen-institute.org/Helicopter-money-to-combat-economic-depression-in-the-wake-of-the-Covid-19.html. Zugegriffen: 13. Aug. 2020.

[CR10] de Boerse (2021). Dax. http://www.boerse.de/historische-kurse/Dax/DE0008469008_seite,2,anzahl,20. Zugegriffen: 2. Febr. 2021.

[CR32] de imsalon (2020). Mehrheit der Friseure entscheidet sich für Preiserhöhung zwischen 10-20. http://imsalon.de/artikel/artikel-detailseite/umfrage-preiserhoehung0. Zugegriffen: 13. Aug. 2020.

[CR18] empirica (2020). empirica Immobilienpreisindex II/2020. Mieten steigen wiederholt langsamer als Kaufpreise. http://www.empirica-institut.de/thema/regionaldatenbank/empirica-immobilienpreisindex. Zugegriffen: 13. Aug. 2020.

[CR19] Europäische Zentralbank (2020a). Our response to the coronavirus pandemic. http://www.ecb.europa.eu/home/search/coronavirus/html/index.en.html. Zugegriffen: 13. Aug. 2020.

[CR20] Europäische Zentralbank (2020b). Statement by the President of the ECB—2 March 2020. http://www.ecb.europa.eu/press/pr/date/2020/html/ecb.pr200302~f2f6113f52.en.html. Zugegriffen: 13. Aug. 2020.

[CR21] Europäische Zentralbank (2020c). What is excess liquidity and why does it matter? http://www.ecb.europa.eu/explainers/tell-me-more/html/excess_liquidity.en.html. Zugegriffen: 13. Aug. 2020.

[CR22] Eurostat (2020a). Euro area annual inflation down to 0.1. http://ec.europa.eu/eurostat/documents/2995521/10294984/2-17062020-AP-EN.pdf/27a39aa1-8a45-10f9-dc06-71d41f235e03. Zugegriffen: 13. Aug. 2020.

[CR23] Eurostat (2020b). Euro area annual inflation down to 0.4. http://ec.europa.eu/eurostat/documents/2995521/10294696/2-30042020-AP-EN.pdf/695df4c4-1a67-bf92-3a0f-69534046cbfe. Zugegriffen: 13. Aug. 2020.

[CR24] Eurostat (2020c). Euro area annual inflation up to 0.4. http://ec.europa.eu/eurostat/documents/2995521/11156763/2-31072020-AP-EN.pdf/c033a89c-da21-8888-d9a1-3bc1d0ce1a6f. Zugegriffen: 13. Aug. 2020.

[CR25] Eurostat (2020d). House prices up by 5.0 % in the euro area—109/2020—8 July 2020. http://ec.europa.eu/eurostat/documents/2995521/11074516/2-08072020-AP-EN.pdf/8f420fb9-6d3b-7a9e-68d7-e6a7b197c20b. Zugegriffen: 13. Aug. 2020.

[CR26] finanzen.net (2020). Ölpreis in USD (WTI)—Historische Kurse. http://www.finanzen.net/rohstoffe/oelpreis/historisch. Zugegriffen: 13. Aug. 2020.

[CR27] Fontane T (1896). Effi Briest.

[CR28] Friedman M (1992). Money mischief: episodes in monetary history.

[CR29] Heise, M. (2020). Chefökonom Heise warnt: Kommt nach Corona doch die Inflation? http://www.finanzen.net/nachricht/private-finanzen/euro-am-sonntag-standpunkt-chefoekonom-heise-warnt-kommt-nach-corona-doch-die-inflation-9106375. Zugegriffen: 13. Aug. 2020.

[CR30] Hochstein, M. (2020). The risks of relying on an inaccurate inflation measure. http://www.allianzgi.com/-/media/allianzgi/globalagi/documents/allianzgi-hochstein-inaccurate-inflation-measure.pdf. Zugegriffen: 22. Feb. 2021.

[CR31] Huber J (2016). Sovereign money: beyond reserve banking.

[CR33] Internationaler Währungsfonds (2020a). World economic outlook, April 2020: the great Lockdown. http://www.imf.org/en/Publications/WEO/Issues/2020/04/14/weo-april-2020. Zugegriffen: 13. Aug. 2020.

[CR34] Internationaler Währungsfonds (2020b). World Economic Outlook, January 2020. http://www.imf.org/en/Publications/WEO/Issues/2020/01/20/weo-update-january2020. Zugegriffen: 13. Aug. 2020.

[CR35] Internationaler Währungsfonds (2020c). World Economic Outlook, October 2020: a long and difficult ascent. http://www.imf.org/en/Publications/WEO/Issues/2020/09/30/world-economic-outlook-october-2020. Zugegriffen: 2. Febr. 2021.

[CR36] Investing.com (2021). World central banks. https://www.investing.com/central-banks. Zugegriffen: 2. Febr. 2021.

[CR37] Jonung, L., & Roeger, W. (2006). The macroeconomic effects of a pandemic in Europe. A model-based assessment. http://ec.europa.eu/economy_finance/publications/pages/publication708_en.pdf. Zugegriffen: 13. Aug. 2020.

[CR38] Leandro, A., & Llorens i Jimeno, E. (2020). The impact of the COVID-19 outbreak on European inflation. http://www.caixabankresearch.com/impact-covid-19-outbreak-european-inflation. Zugegriffen: 13. Aug. 2020.

[CR39] Macrotrends (2020). Gold prices—100 year historical chart. http://www.macrotrends.net/1333/historical-gold-prices-100-year-chart. Zugegriffen: 13. Aug. 2020.

[CR17] De Marcos CD (1961). An inquiry into the nature and causes of international indebtedness. Anales/Mendoza.

[CR40] Märkl, A. (2020). Interview. Corona-Krise: Müssen wir mit einer Inflation rechnen? http://www.b4schwaben.de/b4b-nachrichten/augsburg_artikel,-coronakrise-muessen-wir-mit-einer-inflation-rechnen-_arid,261588.html. Zugegriffen: 13. Aug. 2020.

[CR41] National Health Commission of the People’s Republic of China (2020). Top expert: Disease spread won’t be on scale of SARS. http://en.nhc.gov.cn/2020-01/21/c_75991.htm. Zugegriffen: 13. Aug. 2020.

[CR42] Office for National Statistics (2019). Shrinkflation: how many of our products are getting smaller? http://www.ons.gov.uk/economy/inflationandpriceindices/articles/theimpactofshrinkflationcpihuk/howmanyofourproductsaregettingsmaller. Zugegriffen: 13. Aug. 2020.

[CR43] Organisation für wirtschaftliche Zusammenarbeit und Entwicklung (2020a). Inflation (CPI). https://data.oecd.org/price/inflation-cpi.htm. Zugegriffen: 13. Aug. 2020.

[CR44] Organisation für wirtschaftliche Zusammenarbeit und Entwicklung (2020b). Share prices. https://data.oecd.org/price/share-prices.htm#indicator-chart. Zugegriffen: 13. Aug. 2020.

[CR45] Parker, M. (2006). The impact of disasters on inflation. *ECB Working Paper No. 1982*. http://www.ecb.europa.eu/home/html/index.en.html. Zugegriffen: 13. Aug. 2020.

[CR46] Pastor, L. (2020). Will COVID-19 be followed by inflation? An inter-generational transfer perspective. http://voxeu.org/content/will-covid-19-be-followed-inflation-inter-generational-transfer-perspective. Zugegriffen: 13. Aug. 2020.

[CR47] Röpke W (2008). Economics of the free society.

[CR48] Statista (2020). Monatliche Entwicklung des DAX 2020. http://de.statista.com/statistik/daten/studie/162176/umfrage/monatliche-entwicklung-des-dax. Zugegriffen: 13. Aug. 2020.

[CR49] Statistisches Bundesamt (2020um). Arbeitskosten im 1. Quartal 2020 um 4,3 % höher als im 1. Quartal 2019 – Pressemitteilung Nr. 207 vom 9. Juni 2020. http://www.destatis.de/DE/Presse/Pressemitteilungen/2020/06/PD20_207_624.html. Zugegriffen: 13. Aug. 2020.

[CR50] Warner, J. (2020). Depression or inflation: where will Covid-19 end? http://www.telegraph.co.uk/business/2020/04/12/depression-inflation-will-covid-19-end. Zugegriffen: 13. Aug. 2020.

[CR51] Weltbank (2020a). COVID-19 to plunge global economy into worst recession since World War II. http://www.worldbank.org/en/news/press-release/2020/06/08/covid-19-to-plunge-global-economy-into-worst-recession-since-world-war-ii. Zugegriffen: 13. Aug. 2020.

[CR52] Weltbank (2020b). Domestic credit provided by financial sector (% of GDP). http://data.worldbank.org/indicator/FS.AST.DOMS.GD.ZS. Zugegriffen: 13. Aug. 2020.

[CR53] Weltgesundheitsorganisation (2021). WHO coronavirus disease (COVID-19) dashboard. http://covid19.who.int. Zugegriffen: 2. Febr. 2021.

